# Androgen Receptor Phosphorylation at Serine 308 and Serine 791 Predicts Enhanced Survival in Castrate Resistant Prostate Cancer Patients

**DOI:** 10.3390/ijms140816656

**Published:** 2013-08-13

**Authors:** Pamela McCall, Claire E. Adams, Jennifer M. Willder, Lindsay Bennett, Tahir Qayyum, Clare Orange, Mark A. Underwood, Joanne Edwards

**Affiliations:** 1 Institute of Cancer Sciences, College of Medical, Veterinary and Life Sciences, University of Glasgow, Glasgow G12 8QQ, UK; E-Mails: pamela.mccall@glasgow.ac.uk (P.M.); claire.adams01@gmail.com (C.E.A.); willderj@gmail.com (J.M.W.); lindsaybennett@me.com (L.B.); tahir1@doctors.org.uk (T.Q.); 2 Pathology Department, Southern General Hospital, 1345 Govan Road, Glasgow G51, UK; E-Mail: clare.orange@glasgow.ac.uk; 3 Department of Urology, Royal Infirmary, Glasgow G31 2ER, UK; E-Mail: underwood891@btinternet.com

**Keywords:** androgen receptor, phosphorylation, hormone naive prostate cancer, castrate resistant prostate cancer

## Abstract

We previously reported that AR phosphorylation at serine 213 was associated with poor outcome and may contribute to prostate cancer development and progression. This study investigates if specific AR phosphorylation sites have differing roles in the progression of hormone naïve prostate cancer (HNPC) to castrate resistant disease (CRPC). A panel of phosphospecific antibodies were employed to study AR phosphorylation in 84 matched HNPC and CRPC tumours. Immunohistochemistry measured Androgen receptor expression phosphorylated at serine residues 94 (pAR^94^), 308 (pAR^308^), 650(pAR^650^) and 791 (pAR^791^). No correlations with clinical parameters were observed for pAR^94^ or pAR^650^ in HNPC or CRPC tumours. In contrast to our previous observation with serine 213, high pAR^308^ is significantly associated with a longer time to disease specific death (*p =* 0.011) and high pAR^791^ expression significantly associated with a longer time to disease recurrence (*p =* 0.018) in HNPC tumours and longer time to death from disease recurrence (*p =* 0.040) in CRPC tumours. This observation in CRPC tumours was attenuated in high apoptotic tumours (*p =* 0.022) and low proliferating tumours (*p =* 0.004). These results demonstrate that understanding the differing roles of AR phosphorylation is necessary before this can be exploited as a target for castrate resistant prostate cancer.

## 1. Introduction

Prostate cancer has the highest cancer incidence in males and is the second highest cause of male cancer related mortality [1]. As a whole compared to other cancers, prostate cancer has a relatively low mortality rate. However if you consider only castrate resistant prostate cancer (CRPC), the mortality rate in these men is high and this is the main contributor to prostate cancer having the highest cause of male cancer related mortality.

One of the most challenging problems in the management of prostate cancer is the treatment of patients who no longer respond to hormone therapy and have failed docetaxel chemotherapy. CRPC has evolved from the clinical observation that surgical or medical castration, by blocking the production of testicular androgens, is not curative and virtually all patients have prostate cancer disease recurrence.

Current practice aims to inhibit androgen production and action, thereby reducing stimulation of the androgen receptor (AR). Inhibition of testicular androgen production is achieved surgically (bilateral orchidectomy) or chemically, using gonadotropin-releasing hormone (GnRH) agonists. The action of androgen may be blocked at a peripheral level using anti androgens, which inhibit ligand binding to AR and subsequent activation. Current clinical data implies that the AR is expressed and continues to mediate androgen signalling after failure of this therapeutic approach. However as this does not completely eliminate circulating androgens, sufficient concentrations of dihydrotestosterone may accumulate in tumour cells to maintain AR signalling, especially in the context of up-regulated receptor levels or increased sensitivity of the AR for activation. In addition, ligands of non-testicular origin (adrenal gland or the tumour cells themselves) or ligand-independent activation can contribute to continued AR signalling. To overcome this and to improve patient treatment options, the mechanisms underlying the development of castrate resistance must be fully understood.

The AR not only functions as a transcription factor but also as a core that incorporates multiple extracellular signals. AR effects are influenced by their levels of expression and posttranslational modifications, including phosphorylation on serine [2–4] and tyrosine residues [5], sumoylation [6] and acetylation on lysine residues [7,8]. Many phosphorylation sites at serine and tyrosine residues on the AR have been identified in cell culture systems after different hormonal or growth factor stimulation [2–5,9]. Phosphorylation of AR is stimulated in a ligand dependent manner and also in a ligand independent manner through signal transduction cascades. Phosphorylation of serine residues stabilizes the AR and protects it from proteolytic degradation. In response to androgen-binding, serine 81 is the most frequently phosphorylated site on the AR, giving the highest stoichiometric phosphorylation yield in LNCaP cells treated with androgens. This phosphorylation has been associated with AR stability and transcriptional activity and regulates promoter selectivity [10,11]. It is also associated with prostate cancer cell growth in cell line studies, suggesting some significance of AR Serine 81 phosphorylation in AR function [12]. AR transcriptional activity can be induced by EGF, and is dependent on AR phosphorylation at serines 515 (MAPK consensus site) and 578 (PKC consensus site), which regulates nuclear cytoplasmic shuffling of the AR, through interactions with the Ku-70/80 regulatory subunits of DNA-dependent protein kinase [3]. Phosphorylation of the AR at serine residues 213 and 791 is predicted to be mediated by Akt, which results in increased AR transactivation [13]. We have previously reported that the expression and activation of the PI3K/Akt cascade influences the progression to castrate resistant disease using clinical prostate cancer tumours, where phosphorylation of AR at the Akt consensus site serine 213 was significantly associated with disease progression [14,15]. The aim of the current study was to establish if other AR phosphorylation sites are associated with patient survival and additionally determine if inhibition or activation at these phosphorylation sites could be employed as therapeutic targets. A number of phosphoserine-specific antibodies have been described for the AR [3,16–18] and the availability of these antibodies provides the possibility of screening clinical samples for changes in receptor phosphorylation, which is of utmost importance for a fuller understanding of the role of this chemical modification in prostate cancer disease progression and may be utilized to predict response to abiraterone.

## 2. Results and Discussion

### 2.1. Clinico-Pathological Detail

Clinical data recorded for each patient included age (70, IQR 66–74), prostate specific antigen (PSA) at diagnosis (34.5 ng/mL, IQR 8.5–126.2), PSA at disease recurrence (16 ng/mL, IQR 4.7–39.5) and Gleason grade at diagnosis (7 range 6–9) and at disease recurrence (9 range 8–9). All patients underwent disease recurrence (median time to disease recurrence 2.88 years, IQR 1.83–4.71 years). The median time to death from disease recurrence was 2.1 (IQR 1.02–3.51) years. The median survival time was 5.82 (IQR 3.44–7.35) years. At last follow-up, 9 patients were alive, 53 patients had died of prostate cancer and 21 patients had died of causes not attributed to prostate cancer. Patients in this cohort were diagnosed with locally advanced (51) or metastatic prostate cancer (30) and subsequently received chemical or surgical castration (27 sub capsular bilateral orchidectomy, 55 GnRH analogue, 2 had both). 57 of the 84 patients also received anti androgen therapy, this included all those who received GnRH analogues. Following disease recurrence, 31 patients received radiotherapy; no patients received taxane therapy. Clinical parameters associated with time to disease recurrence, time to death from disease recurrence and disease specific survival are shown in Table 1.

The strength of this patient cohort is the ability to investigate if changes in protein expression, in the transition from hormone naïve to castrate resistant disease are related to clinical outcome measures. Table 2 demonstrates that only two phosphorylation sites (pAR^308^ and pAR^650^ ) exhibited and increase in expression in the transition from hormone naïve to castrate resistant disease (Table 2).

### 2.2. Protein Expression in the Hormone Naïve Tumours

**Figure 1:**
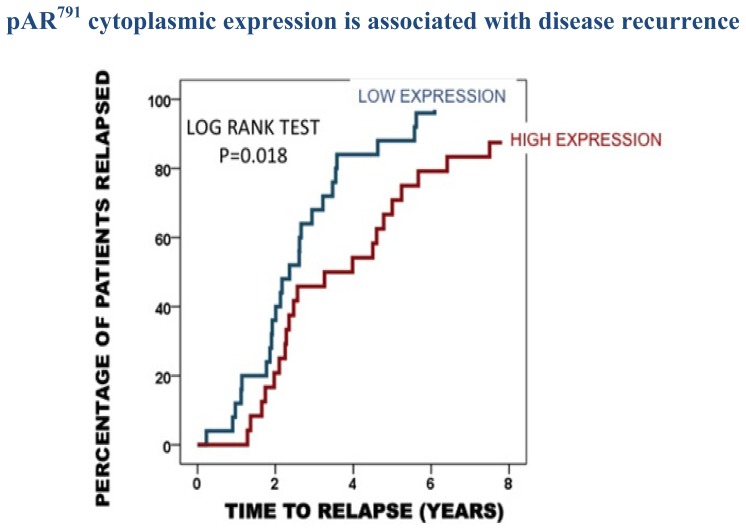
Kaplan Meier plot demonstrates that those patients whose tumours express high pAR^791^ in the cytoplasm (24 patients) have a longer time to disease recurrence than those patients whose tumours exhibit low pAR^791^ expression (25 patients).

To determine if protein expression was linked to time to biochemical relapse, Kaplan-Meier graphs were plotted for the hormone naïve tumours expressing low levels of protein (<median) *versus* high levels of protein (>median) and compared using the log rank test. Expression of pAR^94^, pAR^308^ and pAR^650^ was not associated with clinical parameters in our cohort. However patients with tumours that had high expression of pAR^791^ in the cytoplasm had a significantly longer time to disease recurrence (as measured by time to biochemical relapse) than those patients whose tumours expressed low levels of pAR^791^*p =* 0.018 (Figure 1). The median time to disease recurrence for those with low expression was 2.3 (1.6–3.1) years compared to 3.2 years for high expression (0.8–5.6 years). These patients with high expression were two times more likely to have a longer time to relapse (HR = 2.1 (1.1–3.7) *p =* 0.02). High pAR ^791^ expression was demonstrated to be an independent prognostic marker by Cox regression analysis when compared with the significant clinical parameters PSA at diagnosis, Gleason grade at diagnosis, metastases at diagnosis and radiotherapy (*p =* 0.019).

### 2.3. Protein Expression in the Castrate Resistant Tumours

**Figure 2:**
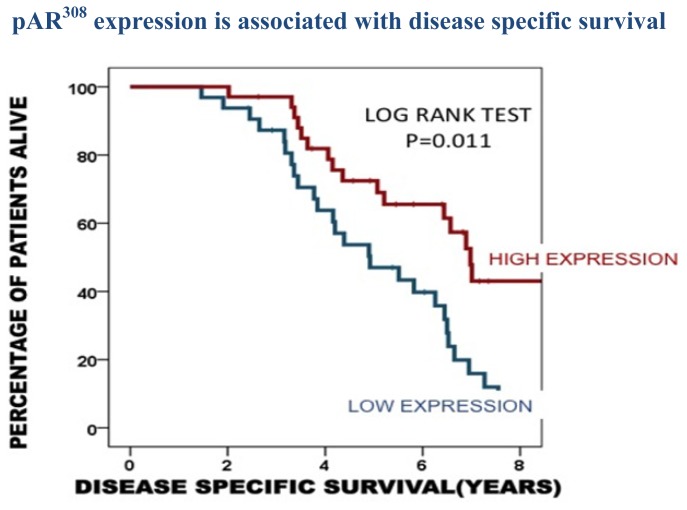
Kaplan Meier plot demonstrates that those patients whose tumours express high nuclear pAR^308^ (34 patients) have a longer disease specific survival than those patients whose tumours exhibit low pAR^308^ expression (32 patients).

To establish if phosphorylated AR expression was linked to time to death from disease recurrence, Kaplan-Meier graphs were plotted for the castrate resistant tumours expressing low levels of phosphorylated AR (<median) *versus* high levels of phosphorylated AR (>median) and compared using the log rank test. Expression of pAR^94^ and pAR^650^ was not associated with clinical parameters in our cohort. However patients whose tumours expressed high levels of nuclear pAR^308^ were observed to have a significantly longer disease specific survival time compared to those patients whose tumours expressed low levels of nuclear pAR^308^ (*p =* 0.011, Figure 2).

**Figure 3:**
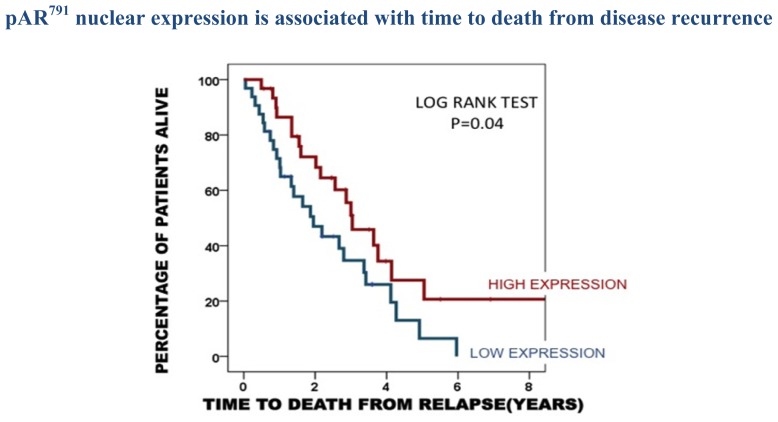
Kaplan Meier plot demonstrates that those patients whose tumours express high pAR^791^ in the nucleus (31 patients) have a longer time to death from disease recurrence than those patients whose tumours exhibit low nuclear pAR^791^ expression (32 patients).

This observation of good prognosis was also observed for pAR^791^. Patients whose tumours expressed high levels of nuclear pAR^791^ were observed to have a significantly longer time to death from disease recurrence compared to those patients whose tumours expressed low levels of nuclear pAR^791^*p =* 0.04 (Figure 3). These patients with high expression were two times more likely to have a longer survival period (HR = 1.9 (1.1–3.4) *p =* 0.04). High pAR^791^ expression was demonstrated to be an independent prognostic marker by Cox regression analysis when compared with significant clinical parameters (*p =* 0.017).

#### 2.3.1. Association of pAR^791^ Expression with Apoptosis and Proliferation

As high expression of pAR^791^ is significantly associated with a longer time to disease recurrence and time to death from disease recurrence, it was investigated if this expression was linked with tumour apoptotic or proliferation index. Kaplan-Meier graphs were then plotted for the castrate resistant tumours expressing low levels of protein (<median) *versus* high levels of protein (>median), stratified by apoptotic or proliferation index and compared using the log rank test.

**Figure 4:**
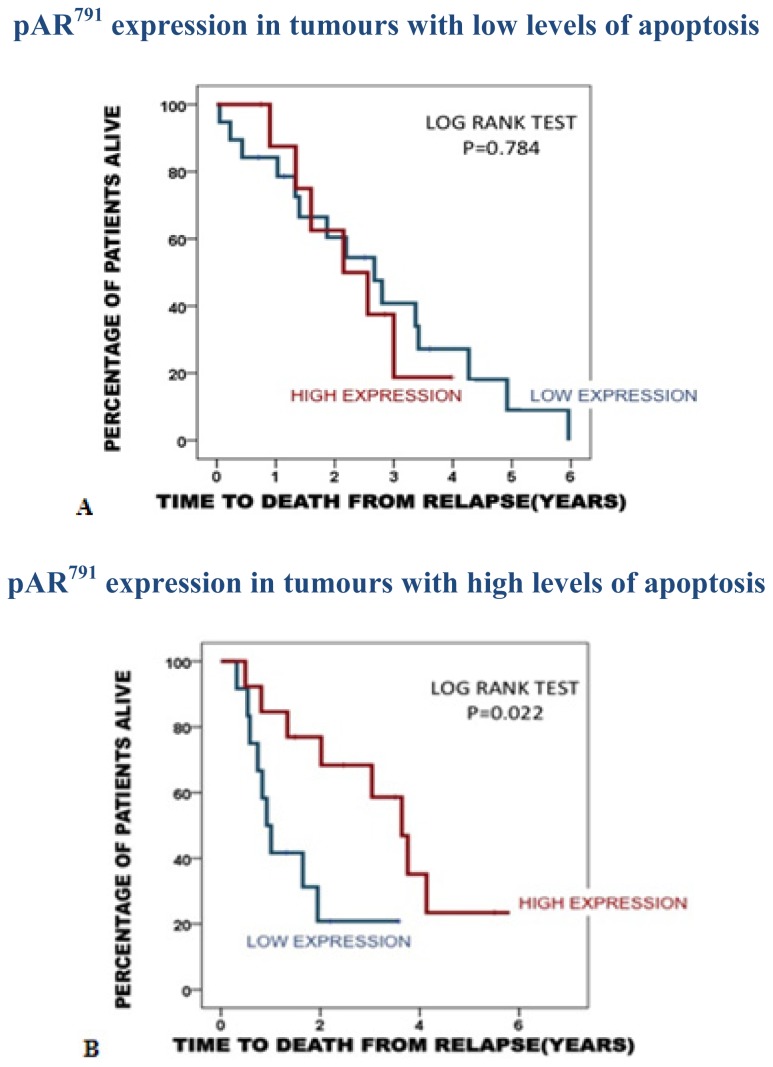
(**A**) Kaplan Meier plot demonstrates that in patients with low apoptotic tumours, pAR^791^ expression is not associated with time to death from disease recurrence (low expression 19 patients, high expression 9 patients.); (**B**) Kaplan Meier plot demonstrates that in patients with highly apoptotic tumours, high pAR^791^ expression is significantly associated with longer time to death from disease recurrence than those with low pAR^791^ expression (low expression 12 patients, high expression 13 patients.).

The association observed for pAR^791^ with a longer time to death from disease recurrence was negated in tumours with low apoptotic index (*p =* 0.784) and attenuated in tumours with a high apoptotic index (*p =* 0.022). The median time to death from disease recurrence for those with low pAR^791^ expression/high apoptosis was 1.5 years (1–2.2) years compared to 3.6 years (2.4–4.9 years) for those with high pAR^791^expression/high apoptosis (Figure 4A,B).

**Figure 5:**
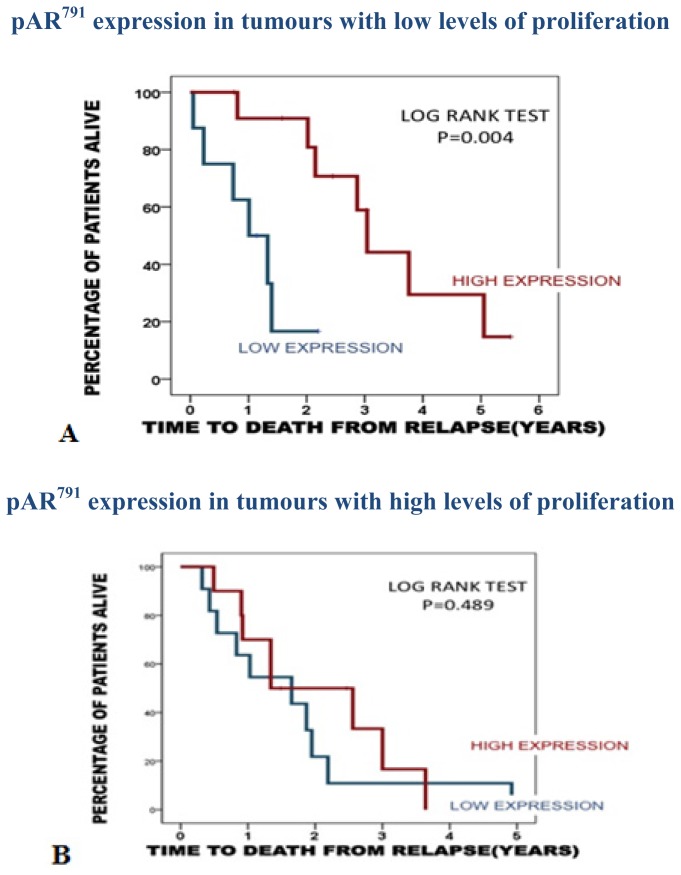
(**A**) Kaplan Meier plot demonstrates that in patients with low proliferating tumours (8 patients), high pAR^791^ expression (12 patients) is significantly associated with longer time to death from disease recurrence than those with low pAR^791^ expression; (**B**) Kaplan Meier plot demonstrates that in patients with high proliferation tumours, pAR^791^ expression is not associated with time to death from disease recurrence (low expression 11 patients, high expression 12 patients).

The association observed for pAR^791^ with a longer time to death from disease recurrence was also attenuated in tumours with low proliferation index (*p =* 0.004) and negated in tumours with a high proliferation (*p =* 0.489). The median time to death from disease recurrence for those with low pAR^791^ expression/low proliferation index was 1.1 years (0.5–1.6) years compared to 3.4 years (2.4–4.3 years) for those with high pAR^791^ expression/low proliferation (Figure 5A,B).

The role of pAR^791^ expression therefore appears greater in slow proliferating, apoptotic tumours. pAR^308^ expression was not associated with either tumour apoptosis or proliferation.

#### 2.3.2. Association of pAR^791^ Expression with AR Gene Amplification

**Figure 6:**
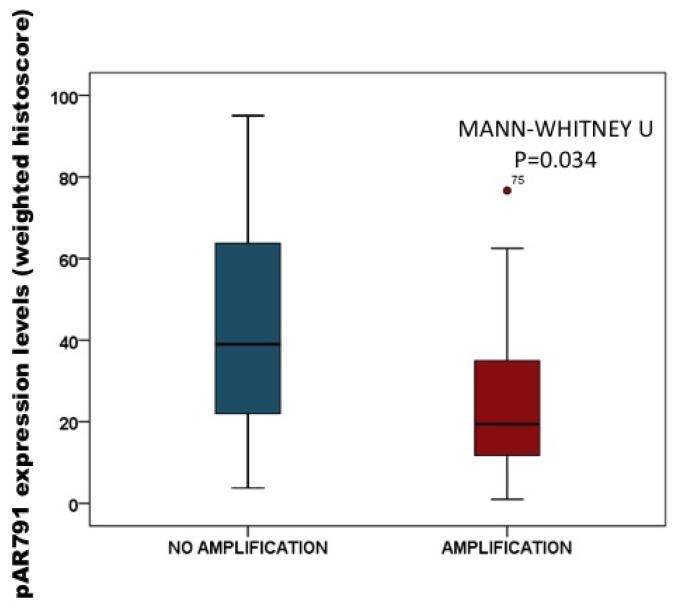
Box plot demonstrates that pAR^791^ expression is significantly lower in patients with AR amplification (non amplification 30 patients, amplification 18 patients).

Data for AR gene amplification from the same cohort of patients was available for analysis. This allowed us to determine if AR amplification was associated with the phosphorylation levels of AR expression in this study. By dividing protein expression into tumours with or without AR amplification, tumours with AR amplification had significantly lower nuclear pAR^791^ expression compared to those without AR amplification (*p =* 0.034) (Figure 6). Median nuclear pAR^791^ expression for castrate resistant tumours with AR amplification is 19 (IQR, 11–35) histoscore units compared to 39 (IQR, 19–69) histoscore units, for tumours that do not have AR amplification.

### 2.4. Discussion

One of the many challenges in the effective management of prostate cancer is the identification of molecular markers capable of predicting disease progression. Castrate resistant disease represents the lethal phenotype of prostate cancer. The main aim of this study was to investigate the effect of androgen receptor phosphorylation in the development and progression of prostate cancer and to determine if it influenced patient outcome. Translating the *in vitro* evidence into the clinical scenario, this study established that phosphorylation at specific serine residues on the AR are involved in the development of castrate resistant disease.

Utilizing this cohort, we have previously reported that phosphorylation of AR at serine 213 is associated with poor prognosis in CRPC [14,15] and it is accepted that in addition to protecting and stabilizing AR, phosphorylation of specific serine residues can influence a ligand-independent induction of transcription by the AR [19,20]. Therefore the ability of signaling cascades to influence AR function may play a significant role in the development and progression of prostate cancer. We, and others, have demonstrated that multiple signal transduction pathways couple with the acquisition of castrate resistant disease [14,15,21–23]. As cell line models studies demonstrate phosphorylation of serines 81, 308, and 650 in response to androgen treatment we hypothesis that these pathways drive kinases to phosphorylate and modulate AR activity. Cell cycle-dependent kinases (Cdk)1, 5 and 11 have been implicated in the phosphorylation of serines 81 and 308 [24–26]. In addition, phosphorylation of serine 81 has been demonstrated to be associated with Cdk9. Cdk9 phosphorylation appears to selectively phosphorylate serine 81 to regulate promoter selectivity [11]. Phosphorylation of serine 308 has been associated with repression of AR activity by cyclin D3/Cdk11 [25]. Additionally, it has been observed using phosphospecific antibodies, that compartmentalization influences the phosphorylation state of AR and that there is a bias for androgen-dependent phosphorylation of serines 81, 256, and 308 in the nucleus and androgen-independent phosphorylation of serine 94 in the cytoplasm; the authors suggest one function of nucleocytoplasmic shuttling is to integrate the signaling environment in the cytoplasm with AR activity in the nucleus [26]. In the current study patients with high levels of nuclear pAR^308^ expression were observed to have a survival advantage over those patients with low expression. This observation is consistent with the previous observation that pAR^308^ is associated with repression of AR activity and combined suggests that phosphorylation at this site may play a role in cell cycle arrest. It was surprising that levels of pAR^308^ expression increased with the development of CRPC disease when levels were associated with better prognosis, we hypothesis that as the disease progresses AR phosphorylation levels increase in response to signaling cascades being up-regulated, however due to the promoter selectivity associated with different phosphorylation sites this does not always result in advantage to the tumour.

Serine 650 is phosphorylated *in vitro* by protein kinase A, protein kinase C and by members of the MAPK family, including the stress-induced kinases p38α and JNK [26]. Although phosphorylation of this residue is well documented, the functional consequences of this modification are a subject of debate. Initial studies reported a modest impairment of transactivation when the serine is mutated to alanine [20]. Yet others have observed no major changes in transactivation activity with the same mutation [27]. Gioeli *et al*. have demonstrated that serine 650 phosphorylation regulates nuclear export, with the non-phosphorylatable S650A mutant receptor retained in the nucleus and a concomitant increase in a target gene expression [25]. In addition it has been reported that serine 650 phosphorylation is a target for protein phosphatase 1, and that it is involved in nuclear export and subsequent receptor turnover [24]. However in the present study, pAR^650^ expression was not associated with clinical outcome measures.

Interestingly, phosphorylation of the AR at serine 791 was observed to be significantly associated with disease progression. High cytoplasmic protein expression (above the median) of pAR^791^ in hormone naïve tumours was observed to be significantly associated with a longer time to disease recurrence. High nuclear expression of pAR^791^ was also significantly associated with a longer time to death from disease recurrence in the castrate resistant tumours. Taken together these observations suggest high levels of AR phosphorylation at serine 791 have a beneficiary effect as patients are living longer. Due to this observation we further investigated whether high expression of pAR^791^ coincided with high apoptotic tumours or low proliferating tumours. Interestingly high pAR^791^ expression was significantly associated with disease specific survival from disease recurrence in patients with highly apoptotic tumours but this relationship was negated in tumours with low levels of apoptosis. Moreover, high pAR^791^ expression was significantly associated with disease specific survival from disease recurrence in patients with low proliferating tumours. Furthermore using the AR gene amplification data from this cohort it was possible to determine whether AR amplification resulted in increased protein expression and it was observed that pAR^791^ expression was significantly lower in castrate resistant patients with AR gene amplification. This result is in agreement with previous findings by Lin *et al.* who have demonstrated that AR is a substrate for Akt which phosphorylates the AR at serine 213 and serine 791. Here the authors observed that only AR phosphorylation at serine 213 could inhibit AR transactivation thus inhibiting AR targets genes such as p21, which modulates androgen/AR-mediated apoptosis. This would provide a rationale along the observations from this study that following phosphorylation by Akt at serine 791, the AR induces cellular apoptosis which in turn leads to an overall survival advantage in a subset of prostate cancer patients. Moreover this study highlights a differential role of Akt phosphorylation on the AR. We have previously observed that pAR^213^ expression is associated with the development of CRPC and observed a strong correlation between pAkt^473^ and pAR^213^ [14]. However, in this study, a weak correlation was observed in the hormone naïve tumours between Akt activation and pAR^791^ (*r*_s_ = 0.426, *p =* 0.034) but no correlation was made in the castrate resistant tumours. In addition when patients were split into AR amplification status it was observed that pAR^213^ expression was significantly higher in AR amplified tumours compared to non amplified tumours (*p =* 0.025) data not shown, whereas pAR^791^ expression is significantly lower in AR amplified tumours compared to non amplified tumours (*p =* 0.034). This data suggests that in the clinical setting another kinase may be responsible for phosphorylating the AR at serine 791.

## 3. Experimental Section

This biological marker study was performed in accordance to the reporting of tumour marker studies criteria (REMARK).

### 3.1. Patients

A total of 84 prostate cancer patients were included in this study, with hormone naïve and castrate resistant tumours from each patient available for analysis (168 tumours in total). All tumours had patient identification removed, and the clinical information database was anonymised. Ethical approval was obtained from the Multicentre Research Ethics Committee for Scotland (MREC/01/0/36) and Local Research and Ethical Committees. Patients were only selected for analysis if they initially responded to hormone treatment (in the form of sub capsular bilateral orchidectomy or LHRH aganosists combined with antiandrogens) but subsequently relapsed (2 consecutive rises in PSA greater than 10%) and had both a hormone naïve and castrate resistant tissue sample available for analysis. Hormone naive tissue was obtained from 18 patients by TRUS guided biopsy and the remaining by TURP and castrate resistant tumours were all obtained by TURP, which was carried out to relieve bladder outflow obstruction.

### 3.2. Tissue Microarray (TMA) Construction

Cores of prostate cancer tissue (4 × 0.6 mm^2^) as identified by a uropathologist were removed from representative areas of high Gleason grade and areas of low Gleason grade of formalin fixed paraffin embedded TURP blocks. These cores were then embedded into recipient array blocks that were constructed in triplicate. Therefore for each patient sample there is an area of high Gleason and low Gleason grade taken in triplicate that accounts for tumour heterogeneity within the sample. Control cores of normal prostate, colon, breast, pancreas, tonsil, kidney, liver and lung tissue were included in each TMA.

### 3.3. Protein Expression Assessment

#### 3.3.1. Antibody Specificity

pAR^791^ antibody was synthesised by CovalAb, Villeurbanne, France. In brief this included phospho peptide synthesis, HPLC and mass spectrometry analysis, conjugation to a carrier protein, pre-immuno bleed, immunisation of 2 specific pathogen free (SPF) rabbits, 3 boosts, 3 bleeds, ELISA titration of all sera and ELISA titration of purified serum. This antibody was made by immunising host animals with conjugated phosphorylated peptide, following the final bleed of the animal the phospho-specific antibodies were selected by affinity purification. Two affinity columns were used; the first column coupled with the non-phosphorylated peptide and the second column with the phosphorylated peptide. The serum then passed through the first column and the non-retaining elute kept and used for the second purification. The eluate was purified on the second column in order to remove antibodies which might recognise the un-phosphorylated peptide. ELISA tests were performed to ensure that the recovered antibody is phospho-specific. Antibody specificity for pAR^94^, pAR^308^ and pAR^650.^ was previously confirmed using a peptide competing assay (Figure S1) [28].

#### 3.3.2. Immunohistochemistry

**Figure 7:**
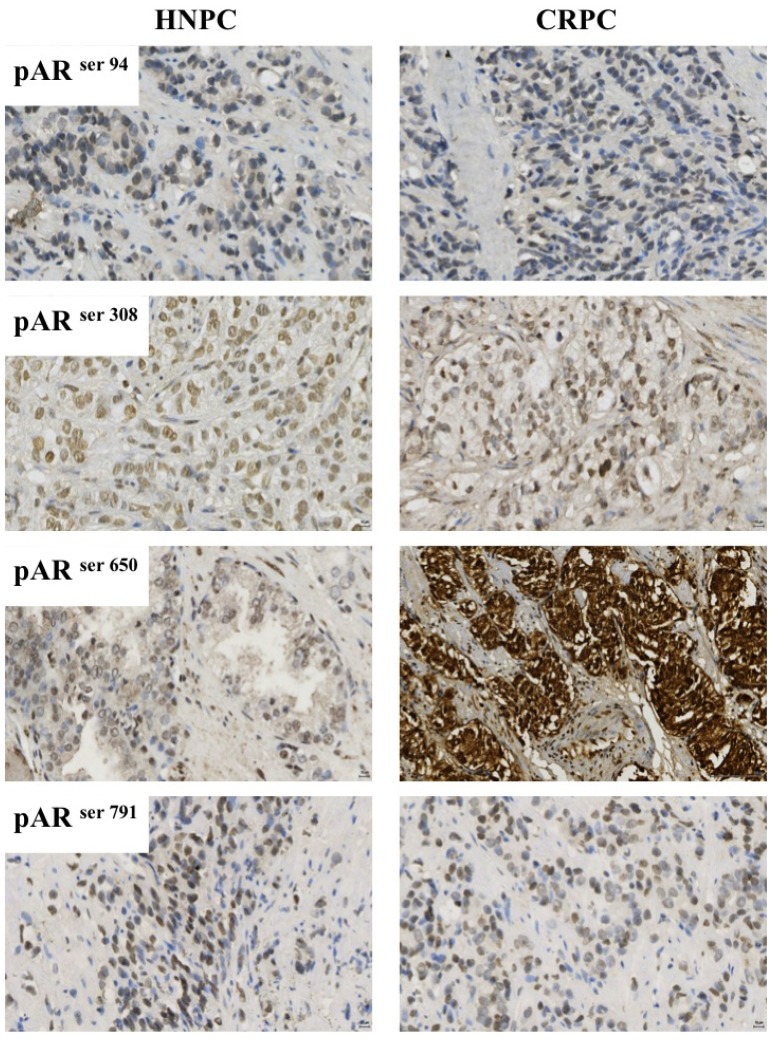
Prostate tumours displaying immunohistochemical staining of pAR^94^, pAR^308^, pAR^650^ and pAR^791^. The bar on each image represents 10 μm

All immunohistochemistry was performed on TMAs with the exception of the TRUS biopsies which were 5 μm, archival formalin fixed, paraffin embedded prostate tumour sections on separate slides. Immunohistochemistry for pAR^94^, pAR^308^, pAR^650^ and pAR^791^ were performed as follows: Sections were dewaxed in xylene and rehydrated through graded alcohol. Antigen retrieval for all proteins was performed using heat treatment under pressure in Tris-EDTA buffer (5 mM Trizma Base, 1 mM EDTA, pH 8) for 5 min. Endogenous peroxidase activity was blocked using 3% hydrogen peroxide and non-specific background staining was blocked using 5% horse serum in TBS for 20 min. pAR^94^ (Abcam, Cambridgeshire, UK), pAR^308^ (Santa Cruz Biotechnology, Santa Cruz, CA, USA), pAR^650^ (Abcam, Cambridgeshire, UK), and pAR^791^ (CovalAb, Villeurbanne, France), antibodies were used at the following concentrations (2 μg/mL, 2 μg/mL, 4 μg/mL, and 50 μg/mL) diluted in Dako antibody diluent (Dako A/S). All antibodies were incubated overnight at 4 °C. Bound antibody complex was visualized using EnVision plus kit (Dako A/S) followed by 3,3-diaminobenzidine tetrahydrochloride (Vector Laboratories). Nuclei were counterstained with haematoxylin before mounting with DPX. A positive (tissue know to express the antigen) and a negative control (tissue known to not express the antigen, and positive control tissue with antibody replaced with isotyped matched antibody) were included in each immunohistochemistry run to exclude the possibility of false-negative and false-positive staining. Examples of staining expression patterns for all markers in both HNPC and CRPC tumours are shown in Figure 7.

Ki67 immunohistochemistry was performed by established protocols in the Department of Pathology, Glasgow Royal Infirmary with appropriate positive and negative controls. Dako anti-Ki-67 (monoclonal mouse anti-human, Ki-67 antigen, clone MIB1, code M7240, DAKO, Glostrup, Denmark) was used at dilution 1:100 for 30 min for immunohistochemistry on a Bond Max automated slide stainer (Leica Microsystems, Wetzlar, Germany) according to the manufacturer’s instructions, with Leica Envision detection system. Slides were lightly counterstained with haematoxylin, dehydrated and mounted with DPX. Apoptotic scoring was performed as per manufacturers instructions using the R & D systems Tunel labeling kit (TdT *in situ* DAB) for light microscopy.

#### 3.3.3. Scoring Method

Tissue staining intensity was scored blind by 2 independent observers using a weighted histoscore method, also known as the Hscore system [29] for phosphorylated AR antibodies. Histoscores were calculated from the sum of (1 × % cells staining weakly positive) + (2 × % cell staining moderately positive) + (3 × % cells staining strongly positive) with a maximum of 300. Proliferation scoring and apoptotic scoring was counting using the point count technique. The numbers of positive and negative tumour cells were counted visually and the percentage of positive cells expressed as a percentage of the total number of cells. The inter-class correlation coefficient (ICCC) for each protein was calculated to confirm consistency between observers and the mean of the two observers’ scores were used for analysis. Changes in staining between pre and post castrate resistant cases were defined as an increase or decrease out with the 95% confidence interval for the difference in inter-observer variation *i.e.*, the mean difference between the histoscores that each observer assigns for protein expression plus 2 standard deviations.

### 3.4. Statistical Analysis

All statistical analysis was performed using the SPSS version 18.0 for Windows. Protein expression data was not normally distributed and is shown as median and inter quartile ranges. Wilcoxon Signed Rank Tests were used to compare protein expression between pre and post castrate resistant tumours. Survival analyses were conducted using Kaplan-Meier method and curves were compared with the log-rank test. Hazard ratios (HR) were calculated using Cox Regression analysis. Multivariate analysis combined any significant proteins of interest with significant clinical parameters to establish if it was independent of these known prognostic markers in influencing patient outcome.

## 4. Conclusions

The findings presented in the current study expand upon our previous work describing the association of phosphorylated serine 213 in prostate cancer tumours and demonstrate that phosphorylation of AR can positively and negatively regulate AR actions. Taken together, this data indicates that phosphorylation of the AR has the potential to regulate AR function in the development and progression of CRPC and it is conceivable that the functional consequences of phosphorylation at individual serine residues maybe gene specific. Further work is necessary to distinguish these possibilities.

## Supplementary Information



## Figures and Tables

**Table 1:** Impact of clinicopathological factors on patient survival.

	Time to disease recurrence	Time to death from disease recurrence	Disease specific survival
Age (<70/>70/unknown)	*p =* 0.210 (56/28)	*p =* 0.247	*p =* 0.246
Gleason (<7/=7/>7/unknown)	*p =* 0.020 (33/39/12)	*p =* 0.165	*p =* 0.261
Metastasis at diagnosis (No/Yes/Unknown)	*p =* 0.007 (66/18)	*p =* 0.016	*p =* 0.0007
PSA at diagnosis (<4/4–10/>10) unknown	*p =* 0.044 (3/13/68)	*p =* 0.246	*p =* 0.261
Metastasis at recurrence (No/Yes/Unknown)		*p =* 0.001 (19/54/13)	*p =* 0.006
PSA at recurrence (<10/10–20/>20) unknown		*p =* 0.019 (37/11/36)	*p =* 0.001

**Table 2:** Protein expression in hormone naïve and castrate resistant tumours: the median histoscore and interquartile range (IQR) for hormone naïve tumours (HNPC) and castrate resistant tumours (CRPC, tumours that have become refractory to hormone treatment) and the *p* value of these values compared using a Wilcoxon sign rank test. ICCC = interclass correlation coefficient. C and N relates to protein cellular location, C (cytoplasm) and N (nucleus). p before a protein indicates that the antibody detects phosphorylated protein and the number following the protein represents the serine site of phosphorylation.

	HNPC (IQR)	CRPC (IQR)	*p* value	ICCC
pAR^94^, C	30 (0–50)	15 (0–36)	*p =* 0.404	0.71
pAR^94^, N	25 (10–39)	20 (9–38)	*p =* 0.158	0.70
pAR^308^, N	135 (110–153)	153 (140–173)	***p =*0.006**	0.77
pAR^650^, C	0 (0–15)	5 (0–11)	*p =* 0.980	0.92
pAR^650^, N	85 (44–110)	104 (74–125)	***p =*0.003**	0.95
pAR^791^, C	20 (0–50)	10 (0–33)	*p =* 0.798	0.92
pAR^791^, N	34 (16–62)	29 (12–54)	*p =* 0.291	0.95
