# Supplementary material for: Androgen Receptor Phosphorylation at Serine 308 and Serine 791 Predicts Enhanced Survival in Castrate Resistant Prostate Cancer Patients
Source: Int J Mol Sci. 2013 Aug 13;14(8):16656–71. doi: 10.3390/ijms140816656 (PMC3759930; doi:10.3390/ijms140816656)
Supplement: Supplementary file 1 [file ijms-14-16656-s001.pdf]

Supplementary Information

Figure S1. Antibody Validation. Peptide competition assays were performed to confirm antibody specificity for each AR serine phosphorylation site. pAR^{S308} (#sc-26406-P Santa Cruz Biotechnology Inc., Dallas, TX, USA) peptide was incubated at a ratio 3:1 for 1 h with each antibody. pAR^{S94} (Protein sequence QQQQQGEDG(pS)PQAH raised in rabbit by EZbiolab Inc., Carmel, IN, USA) and pAR^{S650} (Protein sequence EEGEASSTT(pS)PTEE raised in rabbit by EZbiolab Inc., Carmel, IN, USA) peptides were incubated at ratios of 1:1, 2:1, 500:1 and 100:1 respectively with each antibody overnight at 4 °C. IHC was then performed as described above and results are shown in supplementary data. (**A**) AR 94 blocking peptide experiment. Blocked with ARpSer94 peptide (2:1) (bottom row), positive control top row. Note that staining is not present in ARpSer94 when combined with its corresponding blocking peptide, however staining is maintained across the other phosphorylation sites. Therefore ARpSer94 antibody is specific; (**B**) AR 308 blocking peptide experiment. Blocked with ARpSer308 peptide (3:1) (bottom row), positive control top row. Note that staining is not present in ARpSer308 when combined with its corresponding blocking peptide, however staining is maintained across the other phosphorylation sites. Therefore ARpSer308 antibody is specific; (**C**) AR 650 blocking peptide experiment. Blocked with ARpSer650 peptide (100:1) (bottom row), positive control top row. Note that staining is not present in ARpSer650 when combined with its corresponding blocking peptide, however staining is maintained across the other phosphorylation sites. Therefore ARpSer650 antibody is specific.

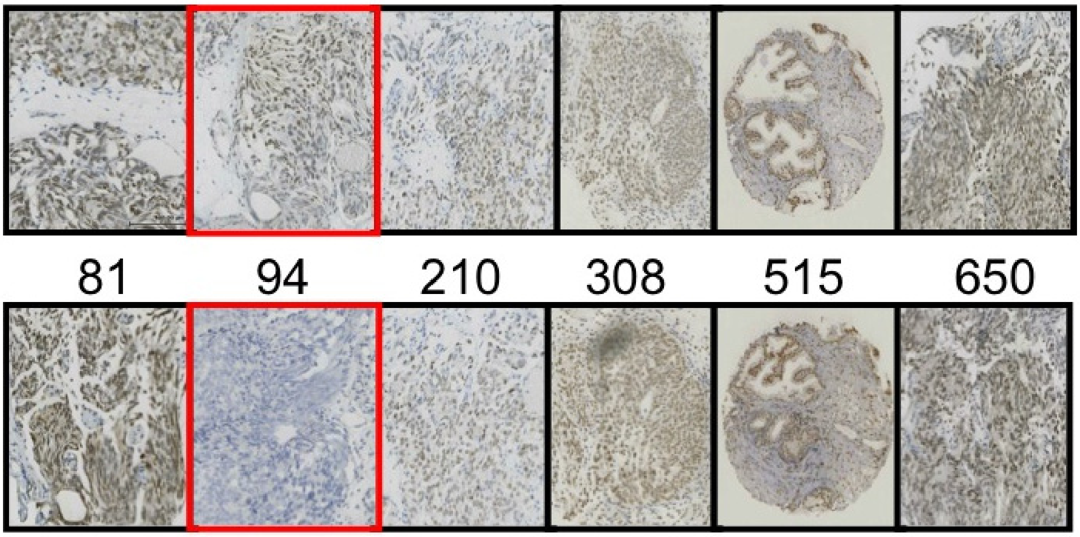

(**A**)

Figure S1. *Cont.*

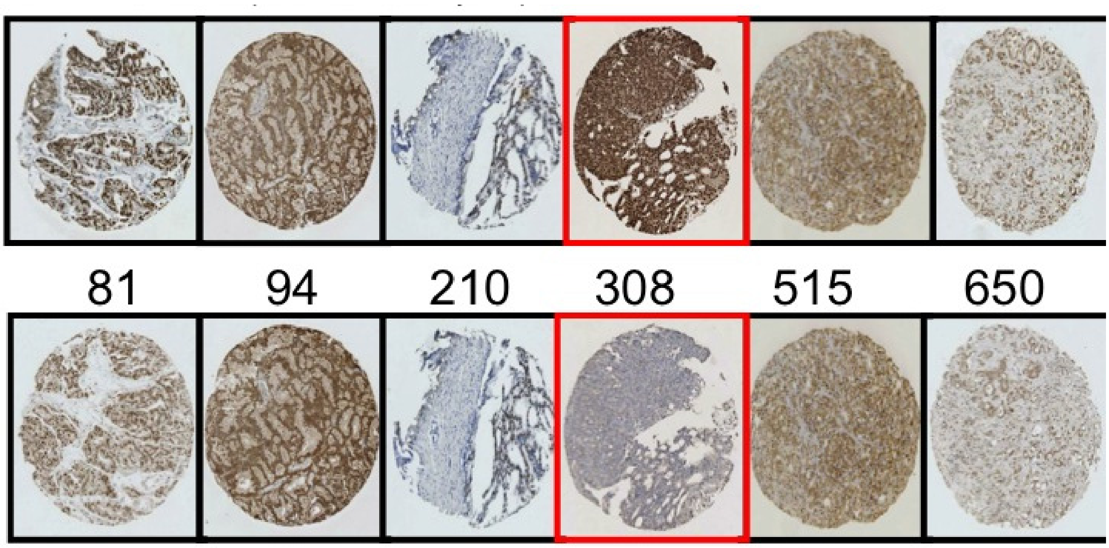

(**B**)

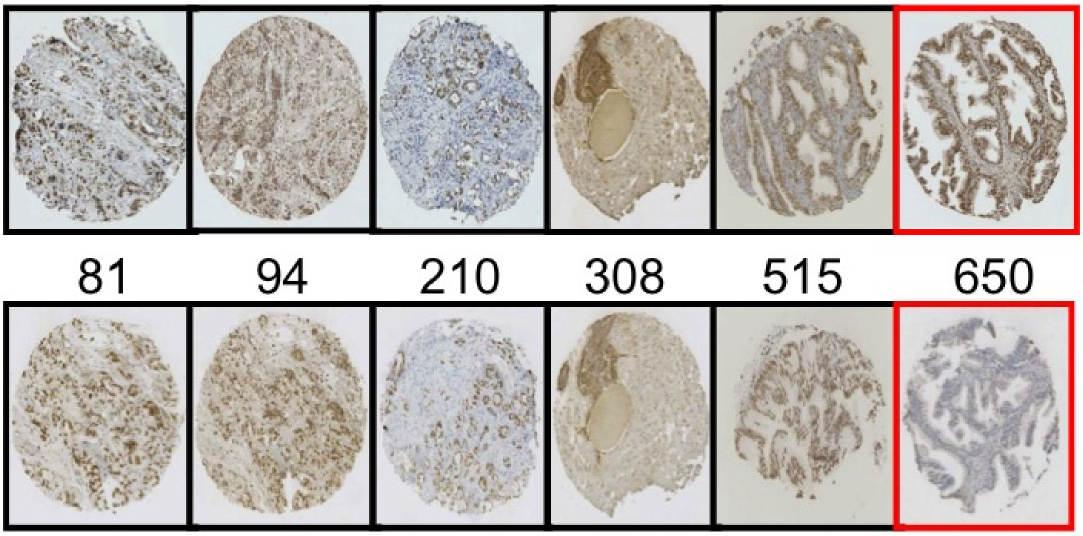

(**C**)

© 2013 by the authors; licensee MDPI, Basel, Switzerland. This article is an open access article distributed under the terms and conditions of the Creative Commons Attribution license (http://creativecommons.org/licenses/by/3.0/).
